# Quantity of alcohol drinking positively correlates with serum levels of endotoxin and markers of monocyte activation

**DOI:** 10.1038/s41598-017-04669-7

**Published:** 2017-06-30

**Authors:** Suthat Liangpunsakul, Evelyn Toh, Ruth A. Ross, Laura E. Heathers, Kristina Chandler, AdePeju Oshodi, Breann McGee, Elizabeth Modlik, Tobyn Linton, Darrin Mangiacarne, Claudie Jimenez, X. Charlie Dong, Li Wang, Wanzhu Tu, David E. Nelson

**Affiliations:** 1 Division of Gastroenterology and Hepatology, Department of Medicine, Indiana University School of Medicine, Indianapolis, IN USA; 2 Roudebush Veterans Administration Medical Center, Indianapolis, Indiana USA; 3 Department of Biochemistry and Molecular Biology, Indiana University School of Medicine, Indianapolis, IN USA; 4 Department of Microbiology and Immunology, Indiana University School of Medicine, Indianapolis, IN USA; 5 Department of Medical and Molecular Genetics, Indiana University School of Medicine, Indianapolis, IN USA; 6 Fairbanks Alcohol and Drug Addiction Treatment Center, Indianapolis, IN USA; 7 Department of Physiology and Neurobiology, and The Institute for Systems Genomics, University of Connecticut, Storrs, CT 06269 USA; 8 Veterans Affairs Connecticut Healthcare System, West Haven, CT 06516 USA; 9 Department of Internal Medicine, Section of Digestive Diseases, Yale University, New Haven, CT 06520 USA; 10 Department of Biostatistics, Richard Fairbanks School of Public health, Indiana University, Indianapolis, IN USA

## Abstract

It is unknown if LPS (lipopolysaccharides) and markers of immune activation, soluble CD14 (sCD14) and CD163 (sCD163) are associated with the quantity of alcohol consumption. 148 subjects were enrolled (97 excessive drinkers (ED) and 51 controls). Time Line Follow-Back questionnaire was used to quantify the amount of alcohol consumed. Serum LPS, sCD14, and sCD163 were measured. Peripheral blood mononuclear cells (PBMCs) were also isolated. Compared to controls, ED had higher total drinks in the past 30 days, higher levels of LPS, sCD14 and sCD163. The levels of serum LPS, sCD14, and sCD163 were higher among ED with recent alcohol consumption (last drink <10 days before enrollment) compared to those without recent drinking. Similar bacterial genome copy numbers were detected in control and ED groups. We found that ethanol primed PBMCs for LPS-induced inflammatory responses. A positive correlation between serum LPS, sCD14, sCD163 and the quantity of alcohol drinking was observed after adjusting for covariates and that abstinence was associated with decline in the levels of LPS, sCD14 and sCd163. We found an increase in the levels of LPS and markers of monocyte activations in ED. Further studies are needed to determine whether these can be used as the biomarkers for excessive alcohol use.

## Introduction

Endotoxin (or lipopolysaccharides, LPS) is the key factor in the pathogenesis of alcohol-induced liver injury in rodent models of acute or chronic alcohol consumption as well as in humans^1, 2^. Excessive alcohol consumption leads to the impairment of intestinal permeability and subsequent bacterial translocation from the gut into systemic circulation. Once in circulation, LPS can bind to the toll-like receptors (TLRs) on many cell types, notably monocytes and macrophages, leading to the activation of inflammatory cytokines^3^; many of which can be used as surrogates for monocyte activation. CD163 is a monocyte scavenger receptor for the hemoglobin-haptoglobin complex^4^. A soluble form of this receptor, sCD163, is shed into the circulation upon monocyte activation^4^. CD14 is a pattern recognition receptor on monocytes. This glycoprotein acts as a co-receptor with TLRs for the detection of bacterial LPS and has membrane-bound (mCD14) and soluble (sCD14) forms^5, 6^. sCD14 appears in the circulation after the shedding of mCD14 during monocyte activation^6^.

A recently published study showed that a single alcohol binge can increase serum endotoxin, disturb innate immune responses, and alter expression of inflammatory cytokines^7^. Interestingly, another report indicated opposite effects of acute and chronic alcohol on LPS-induced monocyte inflammation^8^. To further expand the existing knowledge, this study was conducted to determine: (i) the effect of chronic alcohol drinking on serum LPS levels and markers of monocyte activation (sCD14 and sCD163) in two well characterized cohorts of human subjects with and without excessive alcohol use, (ii) whether excessive alcohol use sensitizes monocyte to LPS stimulation, (iii) the relationship between serum LPS and sCD14/sCD163 in human subjects with and without excessive alcohol use, (iv) the levels of circulating bacteria, using 16s rDNA in subjects with and without excessive alcohol use, (v) the relationship between serum LPS and markers of monocyte activation and the quantity of alcohol consumption during the last 30 days, and (vi) the levels of serum LPS and markers of monocyte activation in response to alcohol abstinence.

## Materials and Methods

### Human subject recruitment

We recruited 97 excessive drinkers (ED) who were seen at Fairbanks Drug and Alcohol Treatment Center (Indianapolis, IN) for alcohol rehabilitation. All met the definition of excessive alcohol use as defined by the NIH/NIAAA; >4 standard drinks in a day (or more than 14 per week) in men and >3 drinks in a day (or more than 7 per week) in women^9^. All reported drinking excessively until the time of enrollment. Fifty-one non-excessive drinkers (controls) were recruited from Richard L. Roudebush Veterans Administration Medical Center. All participants were at least 21 years of age and provided informed consent. Subjects were excluded if they had active and serious medical diseases (such as congestive heart failure, chronic obstructive pulmonary disease, cancer, uncontrolled diabetes, and chronic renal failure); past history of jaundice or complications from portal hypertension, had history of chronic hepatitis B/C infection, had history of any systemic infection within 4 weeks prior to the study; or had history of recent major surgeries within the past 3 months.

To determine the levels of serum LPS and markers of monocyte activation in response to alcohol abstinence, we prospectively recruited 31 subjects with a history of excessive alcohol use who enrolled in a 12-week intensive alcohol treatment program. Blood tests were drawn at baseline (before starting the program) and at weeks 1, 2, 3, 4, 6, 8, 10 and 12 (the end of treatment). All complied with the treatment and none relapsed to excessive alcohol use. The study design and protocol were approved by the Institutional Review Board at the Indiana University Purdue University Indianapolis (IUPUI), Fairbanks Alcohol and Drug Addiction Treatment Center and RLR VAMC Research and Development Program. Further, all methods and experiments were performed in accordance with the relevant guidelines and regulations.

### Data Collection and clinical evaluation

All subjects completed self-administered questionnaires. Demographic data, smoking history (current smokers vs. non-smokers), medical history, and the Alcohol Use Disorders Identification Test (AUDIT-C) were collected. The Time Line Follow-Back (TLFB) questionnaire was used to determine the quantity of alcohol consumption over the 30-day period prior to enrollment. The TFLB was administered in person by trained study coordinators who reviewed the instructions with the subjects prior to administering the questionnaire. The TLFB offers a retrospective report of daily alcohol consumption over the past 30 days; drinks per drinking occasion, and pattern of drinking^10–12^.

### Peripheral blood mononuclear cell (PBMC) isolation

PBMCs were isolated from a subset of excessive drinkers and controls. The complete protocol for PBMC isolation is described in the Supplemental data. Briefly, PBMCs were cultured in buffered RPMI 1640 supplemented with 10% (v/v) heat inactivated fetal bovine serum. Triplicate PBMC cultures (10^6^/ml) were treated with ethanol at the final concentrations ranging from of 6.25 mM-50 mM (1.4 mg–10 mg%) for 2 hrs and then were treated with LPS (*Escherichia coli* L-2630; Sigma Chemical Co., St. Louis, Mo.) 10 ng/ml for 6 hrs. In some experiments, PBMCs were isolated from excessive drinkers, cultured, and were treated with LPS (10 ng/ml). At the end of the experiments, supernatant was collected and the levels of sCD14 and sCD163 were measured.

### Serum endotoxin measurement

Serum endotoxin was measured using the Limulus Amebocyte Lysate QCL-1000^TM^ (Lonza, Walkersville, MD). Gram-negative bacterial endotoxin catalyzes the activation of a proenzyme in the Limulus Amebocyte Lysate. The rate of activation is correlated with the concentration of endotoxin present and was determined photometrically at 405 nM using a microplate method.

### Analysis for markers of monocyte activation, sCD14 and sCD163

Commercially available enzyme-linked immunosorbent assays were used according to the manufacturers’ protocols for measuring sCD14 (R&D System, Cat#DC140, Minneapolis, MN), and sCD163 (MyBioSource, Cat # MBS728094, San Diego, CA).

### Bacterial gDNA isolation and 16S copy number determination by qRT-PCR

Serum samples were re-suspended in 1 ml PBS (final volume), and the resultant suspensions were pelleted by centrifugation for 15 min at 4,000 × *g* at 4 °C, and the pellets were reconstituted in 200 μl PBS. Genomic DNA (gDNA) was isolated using a Qiagen DNeasy blood and tissue extraction kit (Qiagen Inc.) according to the manufacturer’s instructions, eluted in TE buffer, and stored at 4 °C. Reagent only controls were processed in parallel to monitor for reagent contamination. Total bacterial loads in specimens were measured using a quantitative PCR (qPCR) assay based on the TaqMan chemistry, using a broad-range 16 S rRNA gene primer/probe set (forward, 5′-AGAGTTTGATCMTGGCTCAG-3′; reverse, 5′-TTACCGCGGCKGCTGGCAC-3′) and a hydrolysis probe (5′-6-FAM/CCAKACTCCTACGGGAGGCAGCAG/6-TAMRA-3′). Each reaction was performed in triplicate and contained 1 μl of gDNA preparation in a total reaction volume of 10 μl. The cycling conditions were: 95 °C for 2 min, followed by 40 cycles of 95 °C for 15 s, 60 °C for 30 s, 68 °C for 30s. Each 96-well plate contained six serial 10-fold dilutions of plasmid pGEM-16S standards.

### Statistical analysis

Basic descriptive statistics, including frequencies and means, percentages, and standard errors were used to characterize the study subjects. Chi-square test was used to compare values of categorical variables. Student’s t-test or ANOVA were used to compare the values of continuous variables. A multivariate linear regression analysis was used to determine the independent predictors of variables associated with serum endotoxin and markers of macrophage activation. All statistical analyses were performed using SAS 9.3 (Cary, NC). Significance was set at a two-sided *p* < 0.05.

## Results

### Demographic and clinical characteristics of the study cohort

During the study period, 148 subjects were enrolled. The detailed demographics and clinical characteristics of the study cohort are summarized in Table 1. ED were older (41.3 ± 11.6 vs. 31.8 ± 9.3 yrs, p < 0.001), had lower percentage of male gender (67% vs. 88%, p < 0.05), and had higher percentage of current smokers (62% vs. 39%, p < 0.05), compared to non-excessive drinkers (controls). ED had higher AUDIT scores (27.8 vs. 4.8, p < 0.0001) and greater total standard drinks in the past 30 days (242 vs. 15 drinks, p < 0.0001), when compared to controls. They had significantly higher levels of serum aspartate aminotransferase (AST:30 vs. 23 IU/L, p = 0.02), alanine aminotransferase (ALT:29 vs. 24 IU/L, p = 0.01), gamma glutaryl transferase (GGT:89 vs. 35 IU/L, p = 0.001), and %carbohydrate deficient transferrin (%CDT:2.6% vs. 1.5%, p < 0.001). For hematological profiles, ED had lower hemoglobin levels (13.6 vs. 15 g/dL, p = 0.001) and higher levels of mean corpuscular volume (MCV:92 vs. 90, p = 0.01) when compared to controls.

**Table 1:** Demographic and clinical characteristics of the study cohort (N = 148).

Variables	Non-excessive drinkers (N = 51)	Excessive drinkers (N = 97)	p-value
Age (years)	31.8 ± 9.3	41.3 ± 11.6	<0.001
Gender (Male, %)	88	67	<0.05
Race (White, %)	84	80	NS
Smoking status (current smoker, %)	39	62	<0.05
AUDIT scores	4.8 ± 4.6	27.8 ± 4.6	<0.001
Total drinks in the past 30 days, (drinks)	15 ± 15	242 ± 146	<0.0001
BMI (kg/m^2^)	28.7 ± 4.2	27.9 ± 6.2	0.38
Creatinine (mg/dL)	1 ± 0.00	0.9 ± 0.2	0.11
Bilirubin (mg/dL)	0.7 ± 1.3	0.8 ± 0.5	0.68
Albumin (g/dL)	4.1 ± 0.4	3.8 ± 0.4	0.001
Alkaline phosphatase (U/L)	58.2 ± 34.6	59.4 ± 20.2	0.79
AST (U/L)	23 ± 10	30 ± 17	0.02
ALT (U/L)	24 ± 17	29 ± 19	0.01
GGT (U/L)	35 ± 27	89 ± 25	0.001
Hemoglobin (g/dL)	15 ± 1.2	13.6 ± 1.6	0.001
%CDT	1.5 ± 0.8	2.6 ± 1.3	<0.001
WBC (x10^3^ cells/mm^3^)	6.9 ± 2.1	6.5 ± 2.2	0.24
Platelets (x10^3^ cells/mm^3^)	231 ± 55	227 ± 68	0.69
MCV (fL)	90 ± 3.9	92 ± 6.5	0.01

### Comparison of serum LPS in controls and ED

Excessive alcohol use impairs intestinal permeability and promotes bacterial translocation. We next determined the level of serum LPS in controls and ED. LPS in ED was significantly higher than controls (4.7 ± 1.2 vs. 1.6 ± 0.7, EU/ml, p < 0.05).

### Markers of monocyte activations, sCD14 and sCD163 are increased in the serum of excessive drinkers

**Figure 1:**
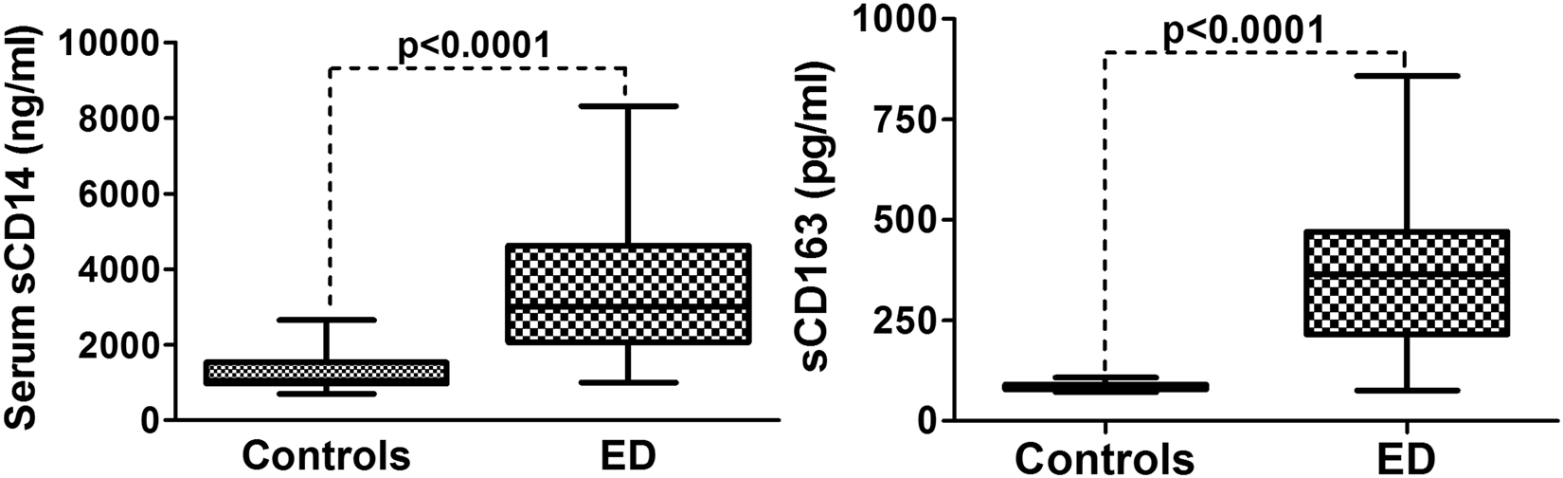
Box plots showed the levels of sCD14 and sCD163 in the serum of controls and excessive drinkers (ED)

Using the serum from subjects in Table 1, we found that ED had higher levels of sCD14 (3454 ± 169 vs. 1267 ± 62.7 ng/ml, p < 0.0001) and sCD163 (359.1 ± 185 vs. 85.2 ± 12.4 pg/ml, p < 0.0001) when compared to controls (Fig. 1).

### Serum endotoxin and markers of monocyte activation among excessive drinkers with and without recent drinking

We stratified excessive drinkers into 2 groups based on the time that the subjects consumed alcohol before the enrollment; excessive drinkers without recent drinking (last drink ≥10 days before the enrollment; this group represents chronic alcohol drinking, N = 41) and those with recent drinking (<10 days before the enrollment; this group represents chronic alcohol with binge drinking, N = 56). We found that the levels of serum LPS, sCD14, and sCD163 were significantly higher in excessive drinkers with recent drinking when compared to those without recent drinking (LPS 5.3 ± 1.2 vs. 3.9 ± 0.8 EU/ml, sCD14 4,030 ± 1722 vs. 2665 ± 1233 ng/ml, and sCD163 4.4 ± 1.6 vs. 2.4 ± 1.4 pg/ml, p < 0.001, Supplementary Table 1).

### Ethanol primes PBMC for LPS-induced inflammatory responses

**Figure 2:**
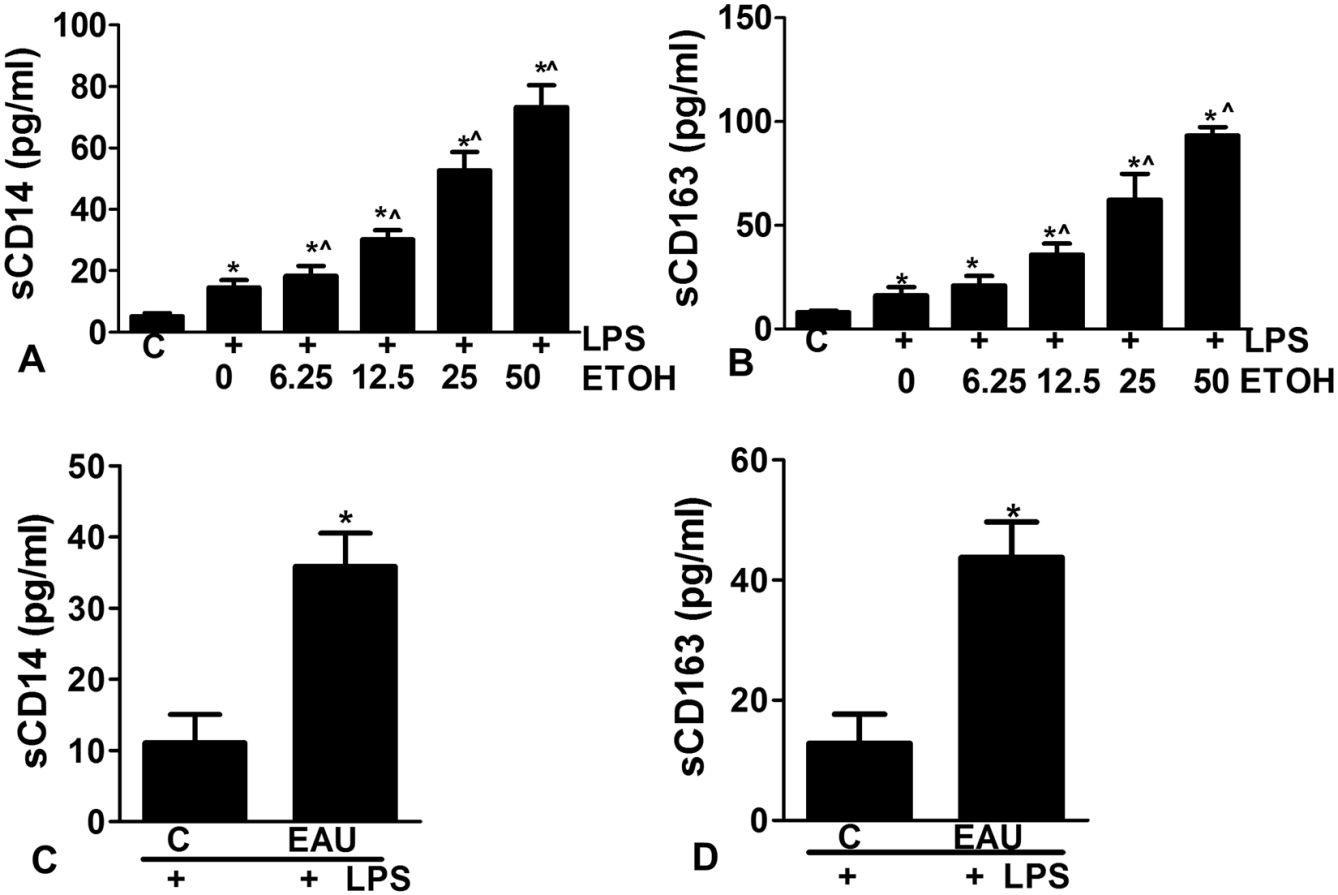
(**A**,**B**) Peripheral blood mononuclear cells (PBMCs) were isolated from healthy controls (**A** and **B**) and pre-treated with ethanol ranging from 6.25 mM–50 mM (1.4 mg–10 mg%) followed by treatment with LPS (10 ng/ml for 6 hrs). Ethanol primes PBMCs for LPS-induced inflammatory responses, as indicated by the increase in the levels of sCD14 and sCD163 in the supernatant in the dose dependent manner. (**C**,**D**) PBMCs were isolated from excessive drinkers and subjected to the stimulation with LPS (10 ng/ml for 6 hrs). sCD14 and sCD163 were significantly increased in supernatants of LPS stimulated PMBCs from subjects with EAU compared to controls. *Compared to controls and ^compared to LPS treatment alone, p < 0.05.

We tested whether alcohol augments the inflammatory response in the monocyte by LPS. Peripheral blood mononuclear cells (PBMCs) were isolated from 8 healthy controls without history of excessive alcohol use. Pre-treatment with ethanol primed PBMCs for LPS-induced inflammatory responses which were indicated by the increase in the levels of sCD14 and sCD163 in the supernatant in an ethanol dose-dependent manner (Fig. 2A,B).

### Excessive alcohol use sensitizes PBMCs to LPS stimulation

We next isolated PBMCs from ED (n = 8, reported average 286 standard drinks in the past 30 days from TLFB) and cultured them as outlined above. The PBMCs were treated with 10 ng/ml LPS for 6 hrs, then supernatant sCD14 and sCD163 levels were measured. sCD14 and sCD163 were significantly increased in supernatants of LPS stimulated PMBCs from subjects with EAU compared to controls. Our results indicate that EAU sensitizes PBMCs to LPS stimulation (Fig. 2C,D).

### Relationship between serum LPS and sCD14/sCD163 in serum of controls and ED

**Figure 3:**
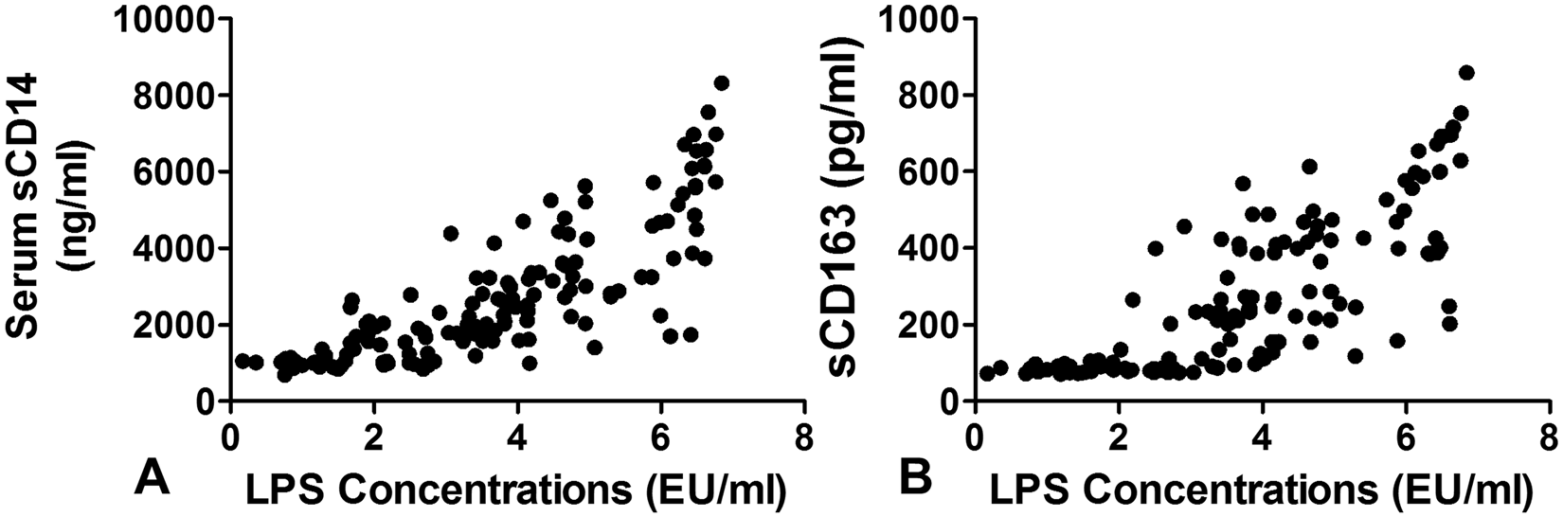
Relationship between serum LPS and sCD14 (**A**) and sCD163 (**B**). The relationship between serum LPS and sCD14/sCD163 was not linear; however, the correlation was evident when the serum LPS > 2 EU/ml which is consistent with the range we observed subjects with excessive drinking.

We next determined the relationship between serum LPS, sCD14 and sCD163. The relationship between serum LPS and sCD14/sCD163 was not linear; however, the correlation was evident when the serum LPS > 2 EU/ml which is consistent with the range we observed subjects with excessive drinking (Fig. 3A,B).

### Levels of circulating bacterial DNA in controls and ED

LPS is the major component of the outer membrane of Gram-negative bacteria so we next tested if the increase in serum LPS in ED correlated with higher numbers of circulating bacteria in this group. To test this we isolated from total gDNA from the serum from the subjects in Table 1 and estimated total bacterial genome copy numbers using a broad-range quantitative PCR assay for bacterial 16S rRNA alleles. Similar bacterial genome copy numbers were detected in the control and ED groups (79.1 ± 316 vs. 60 ± 196 16 S copy number/μl gDNA, p = 0.60).

### Relationship between serum LPS, sCD14, and sCD163 and quantity of alcohol consumption during the last 30 days

**Figure 4:**
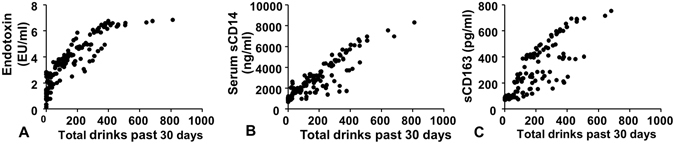
Relationship between serum LPS (**A**), sCD14 (**B**), and sCD163 (**C**) and quantity of alcohol consumption during the last 30 days as measured by timeline follow back. The serum levels of LPS, sCD14, and sCD163 were positively correlated with the quantity of alcohol consumption.

Given that the levels of serum LPS, sCD14 and sCD163 are significantly higher in ED, we next asked if the levels of LPS, sCD14 and sCD163 correlated with the quantity of alcohol consumption, as measured from TLFB. The quantity of recent alcohol consumption over the past 30 days before the enrollment was determined using the TLFB questionnaire. We found the positive correlation between serum LPS, markers of monocyte activation and the quantity of alcohol drinking in the past 30 days (Fig. 4). Additionally, when we analyzed the relationship among the known markers of excessive alcohol use such as AST, ALT, GGT, MCV and %CDT, we found that only the levels of %CDT are associated with the quantity of alcohol drinking (Supplementary Fig. 1). To determine whether the quantity of alcohol consumption was an independent predictor of serum LPS, sCD14, and sCD163, we performed multivariate linear regression analyses controlling for other possible covariates (such as age, gender, race, and smoking status). We found that the quantity of alcohol consumption during the last 30 days was an independent predictor of serum LPS levels and serum markers of monocyte activation (Supplementary Table 2).

### Serum levels of LPS, sCD14, and sCD163 in response to alcohol cessation

**Figure 5:**
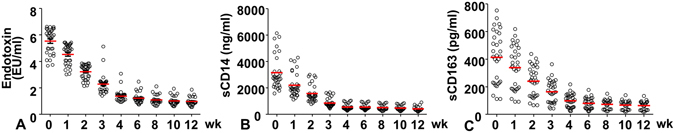
Serum levels of LPS, sCD14, and sCD163 in response to alcohol cessation. Abstinence was associated with significant decline in the levels of LPS (**A**), sCD14 (**B**) and sCd163 (**C**).

The levels of serum LPS, sCD14, sCD163 are increased in response to alcohol drinking. We next hypothesized that the increase in the levels of these markers should persist for a finite time after cessation of drinking and decline after a period of abstinence. We prospectively followed 31 subjects (mean age 39.6 ± 12 yrs, 17 males and the quantity of alcohol consumption in the last 30 days before alcohol rehabilitation 245 ± 150 drinks) with history of excessive alcohol use who enrolled in a 12-week intensive alcohol treatment program. Blood tests were drawn at baseline (immediately before starting the program, wk 0) and at the indicated times as shown in Fig. 5. Abstinence was associated with decline in the levels of LPS, sCD14 and sCd163.

## Discussion

The major findings in our study are the following: (1) ED subjects had higher levels of circulating LPS when compared to controls and the levels of LPS are strongly correlated with the markers of monocyte activation, sCD14 and sCD163, (2) among excessive drinkers, those with recent alcohol drinking history had higher levels of circulating LPS and markers of monocyte activation when compared to those without recent alcohol drinking, (3) excessive alcohol use sensitizes PBMCs to LPS stimulation, (4) despite the increase in the levels of LPS, no differences in the level of bacterial DNA levels were observed between controls and ED subjects, and (5) the positive correlation between serum LPS, markers of monocyte activation and the quantity of alcohol drinking in the past 30 days was observed and their levels were declined upon abstinence.

LPS is a component of the cell walls of gram negative bacteria normally located in the gut^1, 2^. Several studies in animal as well as human have shown that alcohol consumption affects intestinal permeability allowing the LPS from the degrading bacteria crossing into the blood stream^7, 13^. In our large cohort of subjects with excessive alcohol use, we observed an increase in the levels of circulating LPS when compared to controls. More importantly, the patterns of alcohol consumption among excessive drinkers also affected the levels of LPS. Excessive drinkers with recent history of alcohol consumption had significantly higher levels of LPS compared to those without recent alcohol consumption. Circulating LPS can activate immune cells such as monocytes or macrophages by interacting with toll-like receptors (TLRs) on their cell surface; resulting in the release of several cytokines and triggering inflammatory cascades^1, 14^. Among them are sCD163 and sCD14. CD163 is shed from the monocyte/macrophage surface into the circulation as sCD163 upon activation of cell surface Toll-like receptors (TLRs)^4^. CD14 can exist as a glycosylphosphatidylinositol-anchored protein or a soluble form (sCD14) generated by cleavage of the cell surface protein upon monocyte/macrophage activation^6^. We found the serum levels of sCD163 and sCD14 are significantly increased in ED, notably in ED subjects with recent alcohol consumption. These observations are likely due to the increase in the levels of LPS in ED and that excessive alcohol use sensitizes PBMCs in these patients to LPS stimulation, as shown in our data.

One interesting finding from our report is the correlation between the levels of sCD14 and sCD163 and the quantity of alcohol consumption, after adjusting for potential covariates. Such association was not observed with the traditional markers which are normally used to determine the presence of excessive alcohol use (i.e. AST, ALT, GGT, and MCV^15, 16^) with the exception of %CDT. Further studies to determine the diagnostic performance of sCD14 and sCD163 as the biomarkers for excessive alcohol use should be explored.

Despite the increase in the levels of LPS among ED, we however, did not observe an increase in the levels of circulating bacterial DNA in these patients. Our results are contradictory to a previous study in which acute single alcohol binge results in increased serum endotoxin levels and significant increase in serum 16S bacterial rDNA levels at 1, 4 and 24 hours after binge drinking^7^. These findings suggest that drinking patterns may affect the intestinal permeability differently and that in chronic drinkers, the impairment in intestinal permeability may be less than that with binge drinkers leading to only LPS crossing the intestinal barrier but not the bacteria per se.

In summary, we found an increase in the levels of LPS and markers of monocyte activations in those with excessive drinkers. Their levels are correlated with the quantity as well as timing of recent alcohol consumption and declined upon abstinence. Further studies are needed to determine whether these can be used as the biomarkers for excessive alcohol use.

## Electronic supplementary material

Supplementary data
