# Supplementary material for: Quantity of alcohol drinking positively correlates with serum levels of endotoxin and markers of monocyte activation
Source: Sci Rep. 2017 Jun 30;7:4462. doi: 10.1038/s41598-017-04669-7 (PMC5493657; doi:10.1038/s41598-017-04669-7)

Supplementary data

Quantity of alcohol drinking positively correlates with serum levels of endotoxin and markers of monocyte activation

Suthat Liangpunsakul, MD[1,2,3], Evelyn Toh, Ph.D[4], Ruth A. Ross, BS[1], Laura E. Heathers, BS[5], Kristina Chandler, BS[1], AdePeju Oshodi, BS[1], Breann McGee, BS[1], Elizabeth Modlik, BS[6], Tobyn Linton, BS[6], Darrin Mangiacarne, DO, MPH[6], Claudie Jimenez, MD[6], Xiaocheng Dong, PhD[3], Li Wang, PhD[7,8,9], Wanzhu Tu, PhD[10], David E. Nelson, Ph.D[4]

[1]Division of Gastroenterology and Hepatology, Department of Medicine, Indiana University School of Medicine, Indianapolis, IN, [2]Roudebush Veterans Administration Medical Center, Indianapolis, Indiana, [3]Department of Biochemistry and Molecular Biology, Indiana University School of Medicine, Indianapolis, IN, [4]Department of Microbiology and Immunology, Indiana University School of Medicine, Indianapolis, IN, [5]Department of Medical and Molecular Genetics, Indiana University School of Medicine, and [6]Fairbanks Alcohol and Drug Treatment Center, Indianapolis, IN, [7]Department of Physiology and Neurobiology, and The Institute for Systems Genomics, University of Connecticut, Storrs, CT 06269, [8]Veterans Affairs Connecticut Healthcare System, West Haven, CT 06516, [9]Department of Internal Medicine, Section of Digestive Diseases, Yale University, New Haven, CT 06520, [10]Department of Biostatistics, Richard Fairbanks School of Public health, Indiana University, Indianapolis, IN.

Supplementary data

Methods:

PBMC isolation: Blood samples were collected in BD Vacutainer CPT Cell Preparation Tube (cat # 362753) using the standard technique. After collection, the samples were in the upright position at room temperature until centrifugation (speed 1500 RCF for 15 minutes). After centrifugation, half of the plasma was aspirated. Cell layer was collected with a Pasteur Pipette and transferred to a 15 ml size conical centrifuge tube. Cells then were washed with PBS as described in the manufacturer's manual. At the end, freezing medium was added and the PMBCs were aliquoted into cryovials at 5-10X10^6 cell/per tube and then at -80°C freezer till use.

Tables:

Supplementary Table 1: Demographic, clinical characteristics, serum levels of LPS, sCD14, and sCD163 between excessive drinkers with and without history of recent alcohol consumption

Supplementary Table 2: Linear regression analyses on the association between serum levels of LPS, sCD14, and sCD163 and quantity of alcohol consumption during the last 30 days, adjusting for age, gender, race, and smoking status

Figure legends

Supplementary figure 1: Relationship among the known markers of excessive alcohol use (AST, ALT, GGT, MCV and %CDT) and the quantity of alcohol consumption in the last 30 days. Only the levels of %CDT are associated with the quantity of alcohol drinking

Supplementary Table 1: Demographic, clinical characteristics, serum levels of LPS, sCD14, and sCD163 between excessive drinkers with and without history of recent alcohol consumption

Variables	Excessive drinkers without recent alcohol consumption* (N=41)	Excessive drinkers with recent alcohol consumption^ (N=56)	p-value
Age (years)	37.6± 11.6	43.3 ± 12.2	0.02
Gender (Male, %)	60	75	0.18
Race (White, %)	80	80	NS
Smoking status (current smoker, %)	65	57	0.40
AUDIT scores	24.9±7.6	28.0±6.9	0.04
Total Drinks in the past 30 days, drinks	156.8 ± 102	302.9 ± 144	0.02
BMI (kg/m^2)	28.2 ± 7.9	27.6 ± 4.5	0.62
Bilirubin (mg/dL)	0.7 ± 0.3	0.9 ± 0.5	0.12
Albumin (g/dL)	3.7 ± 0.2	3.7 ± 0.4	0.65
Total protein (g/dL)	6.5±0.4	6.6±0.7	0.51
AST (U/L)	38 ± 5.4	37 ± 4.3	0.88
ALT (U/L)	41 ± 6.8	35 ± 4.1	0.01
Hemoglobin (g/dL)	13 ± 1.6	13 ± 1.5	0.37
WBC (x10^{3}cells/mm^3)	6.2 ± 2.4	6.7 ± 1.8	0.27
Platelets (x10^{3}cells/mm^3)	231 ± 72	224 ± 66	0.62
Neutrophil counts (x10^{3}cells/mm^3)	3.2±1.0	4.7±2.1	<0.001
LPS (EU/ml)	3.9±0.8	5.3±1.2	<0.001
sCD14 (ng/ml)	2,665.4±1,233.4	4,030.5±1,722.7	<0.001
sCD163 (pg/ml)	242.8±139	444.2±162	<0.001

* Excessive drinkers with last drink ≥ 10 days before the enrollment
^ Excessive drinkers with last drinks within 10 days before the enrollment

Supplementary Table 2: Linear regression analyses on the association between serum levels of LPS, sCD14, and sCD163 and quantity of alcohol consumption during the last 30 days, adjusting for age, gender, race, and smoking status

Independent variable: Serum LPS				
	Univariate		Multivariate	
**Variables**	**Parameter estimate**	**p-value**	**Parameter estimate**	**p-value**
Age	0.01	0.10	-00006	0.32
Sex	-0.49	0.07	0.01	0.92
Race	-0.04	0.75	-0.008	0.91
Smoking status	-0.20	0.43	-0.14	0.34
Quantity of alcohol consumption in the past 30 days	0.007	<0.0001	0.007	<0.0001
**Independent variable: Serum sCD14**				
	**Univariate**		**Multivariate**	
**Variables**	**Parameter estimate**	**p-value**	**Parameter estimate**	**p-value**
Age	35.7	0.009	5.18	0.49
Sex	-454.1	0.22	217.7	0.29
Race	-14.3	0.93	-5.1	0.95
Smoking status	-138.2	0.69	-34.7	0.85
Quantity of alcohol consumption in the past 30 days	9.75	<0.0001	9.78	<0.001
**Independent variable: Serum sCD163**				
	**Univariate**		**Multivariate**	
**Variables**	**Parameter estimate**	**p-value**	**Parameter estimate**	**p-value**
Age	0.03	0.02	0.005	0.62
Sex	-0.60	0.13	0.01	0.96
Race	0.03	0.89	0.06	0.65
Smoking status	-0.25	0.50	-0.13	0.59
Quantity of alcohol consumption in the past 30 days	0.009	<0.0001	0.009	<0.001

Supplementary Figure 1

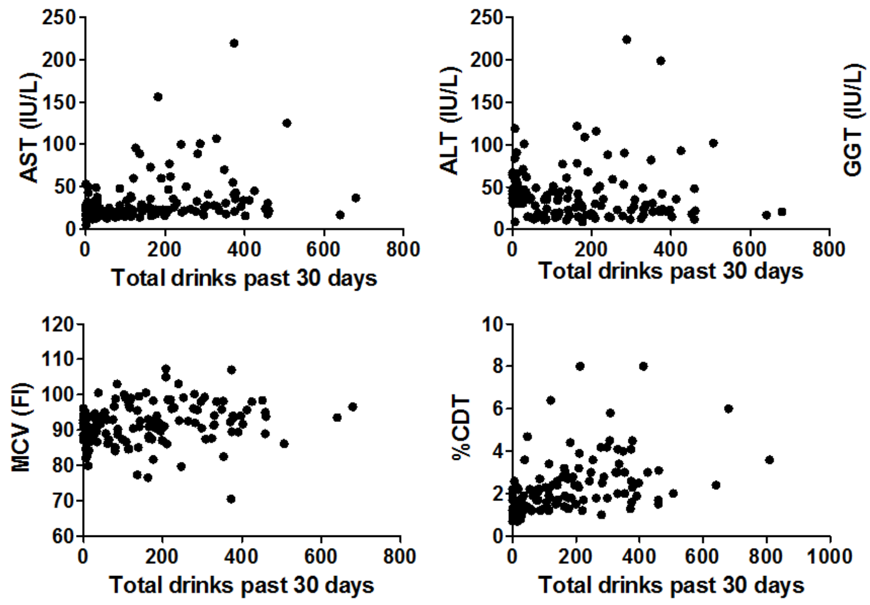

Supplement: Supplementary file 1 — Supplementary data [file 41598_2017_4669_MOESM1_ESM.pdf]
